# Astrocytes display cell autonomous and diverse early reactive states in familial amyotrophic lateral sclerosis

**DOI:** 10.1093/brain/awab328

**Published:** 2022-01-19

**Authors:** Doaa M Taha, Benjamin E Clarke, Claire E Hall, Giulia E Tyzack, Oliver J Ziff, Linda Greensmith, Bernadett Kalmar, Mhoriam Ahmed, Aftab Alam, Eric P Thelin, Nuria Marco Garcia, Adel Helmy, Christopher R Sibley, Rickie Patani

**Affiliations:** 1 Department of Neuromuscular Diseases, Queen Square Institute of Neurology, University College London, London WC1N 3BG, UK; 2 The Francis Crick Institute, London NW1 1AT, UK; 3 Zoology Department, Faculty of Science, Alexandria University, Alexandria 21511, Egypt; 4 Division of Neurosurgery and Wolfson Brain Imaging Centre, Department of Clinical Neurosciences, University of Cambridge, Cambridge CB2 0QQ, UK; 5 Institute of Quantitative Biology, Biochemistry and Biotechnology, School of Biological Sciences, University of Edinburgh, Edinburgh EH8 9JZ, UK; 6 Simons Initiative for the Developing Brain, University of Edinburgh, Hugh Robson Building, George Square, Edinburgh EH8 9XD, UK; 7 Centre for Discovery Brain Sciences, University of Edinburgh, Hugh Robson Building, George Square, Edinburgh EH8 9XD, UK; 8 Euan MacDonald Centre for MND Research, University of Edinburgh, Edinburgh, EH16 4SB, UK

**Keywords:** cell autonomous, amyotrophic lateral sclerosis (ALS), reactive transformation, astrocytes, diversity

## Abstract

Amyotrophic lateral sclerosis is a rapidly progressive and fatal disease. Although astrocytes are increasingly recognized contributors to the underlying pathogenesis, the cellular autonomy and uniformity of astrocyte reactive transformation in different genetic forms of amyotrophic lateral sclerosis remain unresolved.

Here we systematically examine these issues by using highly enriched and human induced pluripotent stem cell-derived astrocytes from patients with *VCP* and *SOD1* mutations.

We show that *VCP* mutant astrocytes undergo cell-autonomous reactive transformation characterized by increased expression of complement component 3 (C3) in addition to several characteristic gene expression changes. We then demonstrate that isochronic *SOD1* mutant astrocytes also undergo a cell-autonomous reactive transformation, but that this is molecularly distinct from *VCP* mutant astrocytes. This is shown through transcriptome-wide analyses, identifying divergent gene expression profiles and activation of different key transcription factors in *SOD1* and *VCP* mutant human induced pluripotent stem cell-derived astrocytes. Finally, we show functional differences in the basal cytokine secretome between *VCP* and *SOD1* mutant human induced pluripotent stem cell-derived astrocytes.

Our data therefore reveal that reactive transformation can occur cell autonomously in human amyotrophic lateral sclerosis astrocytes and with a striking degree of early molecular and functional heterogeneity when comparing different disease-causing mutations. These insights may be important when considering astrocyte reactivity as a putative therapeutic target in familial amyotrophic lateral sclerosis.

## Teaser

Taha *et al*. study astrocyte reactive transformation in ALS using human stem cell models, and show that this can occur cell autonomously. They also reveal a striking, previously unrecognized, molecular and functional heterogeneity between astrocytes derived from iPSCs with genetically divergent ALS-causing mutations (in *VCP* and *SOD1* genes).

## Introduction

Amyotrophic lateral sclerosis (ALS) is a devastating neurodegenerative disease affecting upper and lower motor neurons, resulting in muscle paralysis and ultimately death due to respiratory failure. Ninety per cent of ALS cases are sporadic while the remaining 10% are familial. Studying defined genetic causes of ALS arguably represents a more experimentally tractable approach to understanding disease mechanisms. Between 1% and 2% of familial ALS cases are caused by autosomal dominant mutations in valosin-containing protein (*VCP*/p97), a ubiquitously expressed hexameric protein of the AAA (ATPase associated with diverse cellular activities) family.^1^ VCP/p97 is a multifunctional protein, with crucial roles in maintaining proteostasis through binding to ubiquitinated proteins and facilitating their proteasomal degradation.^2^ Wild-type TDP-43 mislocalization and aggregation together form pathological hallmarks in most ALS cases,^3^ including *VCP*-related ALS.^1^ Conversely, mutations in superoxide dismutase 1 (*SOD1*), which comprise 20% of familial ALS cases, are among the minority that do not typically exhibit TDP-43 mislocalization.^4^ It can therefore be reasoned that *VCP* and *SOD1* mutations are pathologically representative of most recognized distinct states within ALS.

Astrocytes respond in a graded and context dependent manner to a wide spectrum of insults to the nervous system such as traumatic brain injury, spinal cord injury, stroke, inflammation and neurodegenerative diseases.^5^ Astrocyte reactive transformation involves the rapid induction of gene expression that results in complex morphological and functional changes. These changes may include upregulation of complement component 3 (C3).^6,7^ Divergent astrocyte reactive states have been shown when comparing inflammation with stroke.^8^ Recent work suggests that astrocytes can undergo protective or deleterious reactive changes, characterized by distinct gene expression signatures.^6,9^ Potential triggers for the reactive transformation of astrocytes include the release of cytokines (TNF-α, IL-1α) and complement (C1q) by neighbouring cells. Such reactive transformation is associated with perturbed astrocyte homeostatic functions, including their neuroprotective capacity^6^ and cytokine secretion profile.^10^

Astrocytes are increasingly recognized as playing fundamental roles in ALS pathogenesis.^11^ Ourselves and others have previously used human stem cell models to demonstrate non-cell-autonomous mechanisms of disease mediated by SOD1-mutant astrocytes in ALS.^9^ Additionally, we have reported a *VCP* mutant astrocyte survival phenotype compared to control counterparts.^12^ Against this background, we sought to address whether human induced pluripotent stem cell (hiPSC)-derived ALS astrocytes could undergo deleterious reactive transformation cell autonomously. We additionally sought to address whether astrocytes carrying different ALS-causing mutations exhibit a uniform reactive state or whether mutation-specific molecular heterogeneity exists. Through RNA-sequencing (RNA-seq), quantitative immunocytochemistry (qICC) and functional secretome assays, we uncover striking molecular diversity when comparing *VCP* and *SOD1* mutant hiPSC-derived astrocytes. Our findings demonstrate that ALS astrocytes can undergo cell autonomous reactive transformation and that ALS-related early reactive transformation represents a more diverse set of cellular states than previously recognized. These findings have potential implications for therapeutic strategies in ALS that aim to target the harmful reactive transformation of astrocytes.

## Materials and methods

Detailed methods are provided in the Supplementary material.

### HiPSC culture and astrocyte differentiation

HiPSCs were maintained using standard protocols. Astrocyte differentiation was carried out as described previously.^9^ See the Supplementary material for more detailed information.

### Data availability

Study generated transcriptomic data have been deposited in Array Express with accession code E-MTAB-10916. C9ORF72 mutant astrocyte datasets were accessed from accession code GSE142730.

## Results

### HiPSC-derived astrocytes undergo reactive transformation in response to pro-inflammatory cues

**Figure 1:**
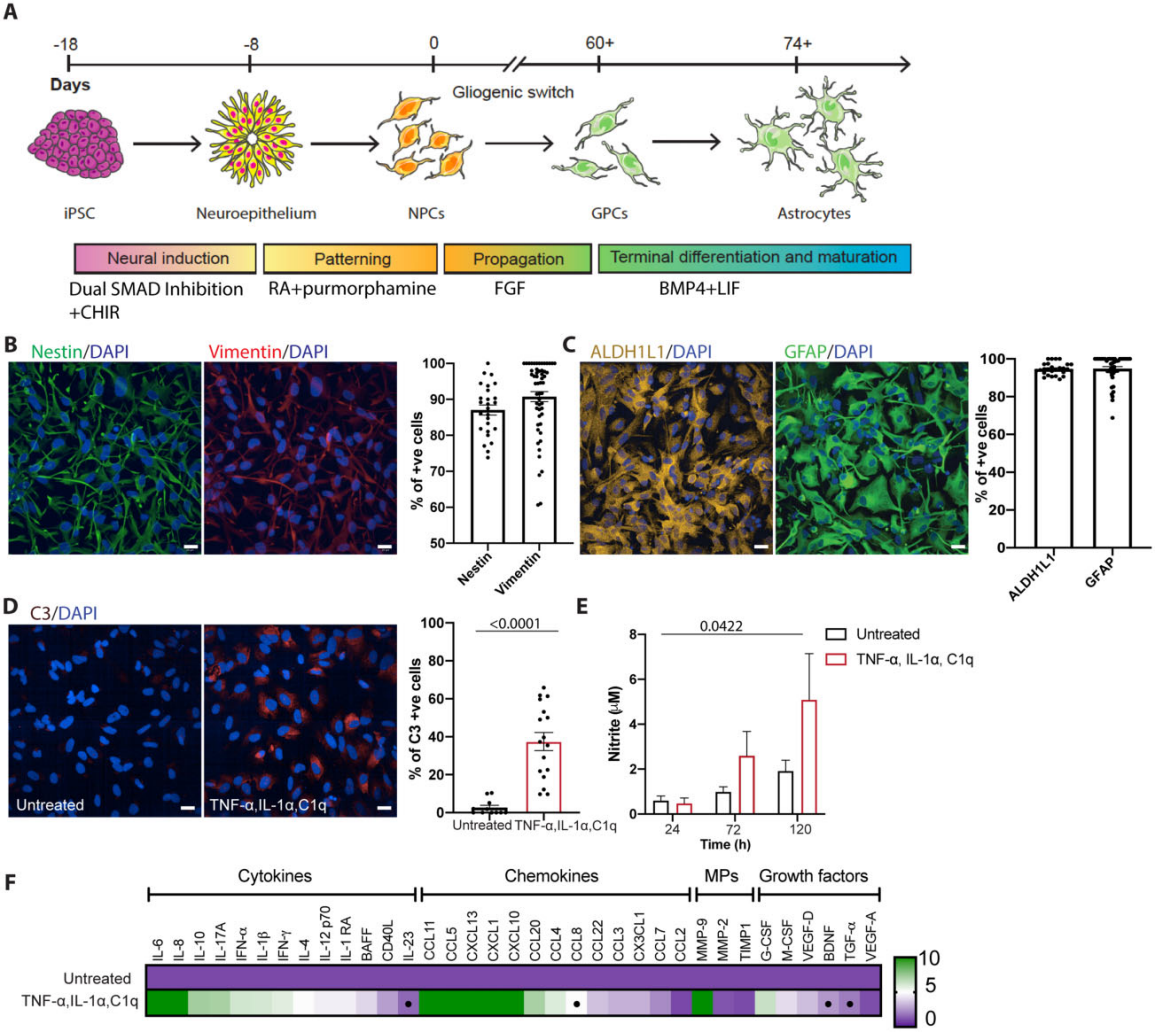
**HiPSC-derived astrocytes undergo reactive transformation in response to pro-inflammatory factors.** (**A**) Schematic depicting hiPSC directed differentiation strategy for producing ventral spinal cord astrocytes. (**B**) Representative immunofluorescence images of glial precursor cells (GPCs > Day 60 FGF) and the percentage of cells immunofluorescent for the neural progenitor marker nestin (green; *n* = 2), and the glial progenitor marker vimentin (red; *n* = 3). (**C**) Representative images and quantification of the percentage of cells immunofluorescent for ALDH1L1 (yellow; *n* = 2) and GFAP (green; *n* = 2). (**D**) Immunofluorescence images of TNF-α, IL-α and C1q treated astrocytes immunolabelled for neurotoxic astrocyte marker C3 (red; *n* = 2) and the percentage of C3-positive cells. Unpaired *t*-test. (**E**) Griess assay of astrocyte conditioned media from TNF-α, IL-α and C1q treated astrocytes at 24, 72 or 120 h. Data-points for the Griess assay are the technical repeats (three wells/line) of two lines in each group. Mixed model ANOVA with *post hoc* Tukey tests. (**F**) Luminex multiplex immunoassay (Thermo Fisher). Data-points for the Luminex multiplex assay are the technical repeats (two wells/line) of two lines in each group. Student’s *t*-test. Data-points of the immunofluorescence image quantification are the number of technical repeats (fields) used in each experiment. Data are presented as mean ± standard error of the mean (SEM). Scale bar = 20 μm. CHIR = CHIR99021; RA = retinoic acid.

We used our previously published directed differentiation paradigm to generate highly enriched (>90%) populations of hiPSC-derived astrocytes^9^ (Fig. 1A). We first confirmed neural and glial precursor identity using qICC for nestin (87.04 ± 1.388%, 26 fields) and vimentin (90.76 ± 1.404%, 54 fields) (Fig. 1B). To assess the enrichment of astrocytes following differentiation of glial precursors, we performed qICC for ALDH1L1, a pan astrocytic marker^13^ and GFAP, one of the principal intermediate filaments in mature astrocytes. The cultured cells expressed ALDH1L1 (94.67 ± 0.6112%, 27 fields) and GFAP (94.85 ± 1.088%, 45 fields) confirming their robust differentiation into astrocytes (Fig. 1C). To ascertain whether our model was suitable to study astrocyte reactivity, we attempted to induce reactive transformation in hiPSC-derived control astrocytes by treatment with established pro-inflammatory factors, TNF-α, IL-1α and C1q,^6,7^ using C3 protein expression as an initial readout. C3 expression was significantly higher in the treated astrocytes [treated control = 37.47 ± 4.739% (17 fields) versus untreated control = 2.670 ± 1.215% (11 fields); *P* < 0.0001; Fig. 1D]. We next addressed whether ALS motor neurons can induce C3 expression in control astrocytes by treatment with motor neuron conditioned medium (MNCM) from *VCP* mutant or *SOD1* mutant lines compared to MNCM from control lines, differentiated using our established protocol.^12^ We found no significant increase in C3 expression in control astrocytes treated with either control or mutant motor neuron conditioned media (Supplementary Fig. 1). We next examined whether reactive transformation of hiPSC-derived astrocytes induced functional changes. Noting that pro-inflammatory stimuli induce nitric oxide synthase (iNOS) in astrocytes and cause the release of nitric oxide (NO),^14^ we used the colorimetric Griess assay to measure NO release into the media. We observed a significant increase in NO release by astrocytes treated with the aforementioned pro-inflammatory factors for 120 h (*P* = 0.0422; Fig. 1E). As further functional characterization, we examined the supernatant for secondary cytokine, chemokine, metallopeptidase and growth factor release, which confirmed significant changes in the reactive astrocytes when compared to untreated control astrocytes, including IL-23, CCL8, BDNF and TGF-ɑ (Fig. 1F). These data together indicate that combinatorial treatment with TNF-α, IL-1α and C1q can robustly induce reactive transformation in enriched populations of hiPSC-derived astrocytes from both molecular and functional perspectives.

### HiPSC-derived *VCP* mutant astrocytes undergo cell-autonomous reactive transformation

**Figure 2:**
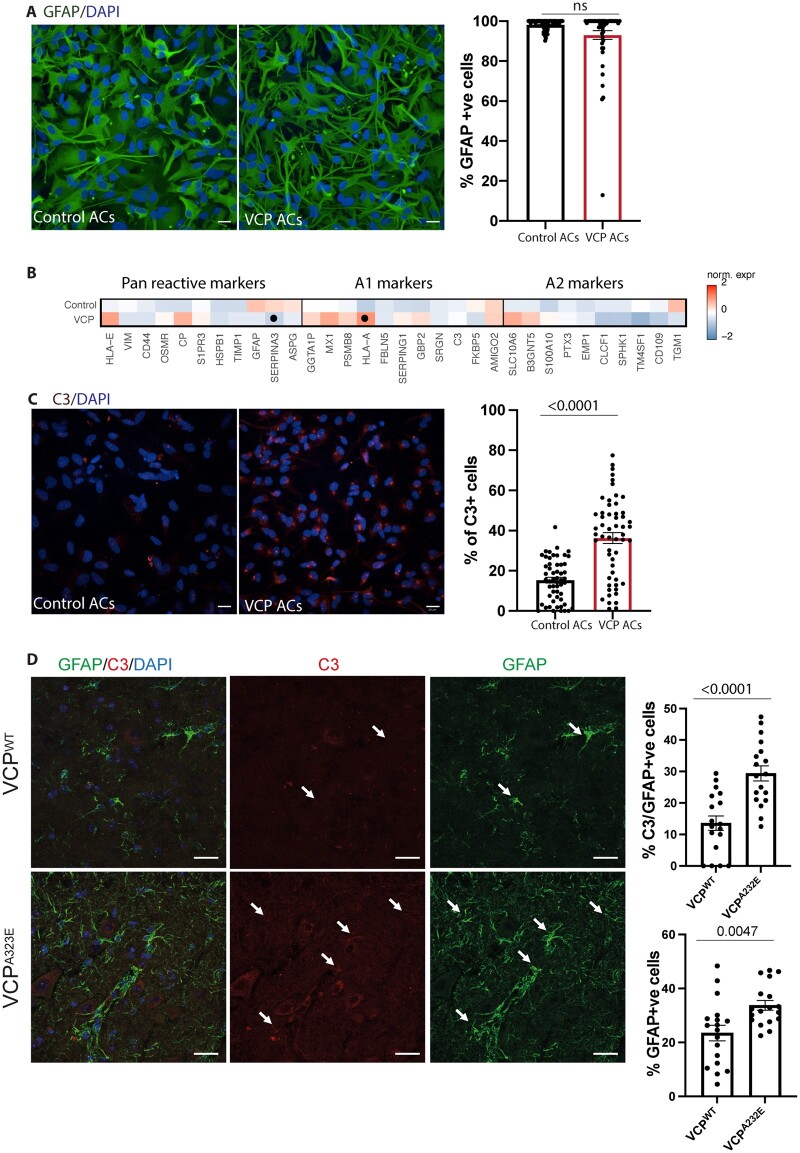
**Cell-autonomous reactive transformation of *VCP* mutant astrocytes.** (**A**) Representative images and quantification of the percentage of cells immunofluorescent for GFAP (green). Mann-Whitney test. Two experimental repeats of two control and two *VCP* mutant lines as a minimum. (**B**) Heat maps of pan reactive, A1 and A2 markers in control and *VCP* astrocytes. Gene expression data represent standard deviation (SD) from mean of the variance stabilized values across control and *VCP* samples. Points represent significantly increased transcripts as determined with the Wald test and following correction for multiple testing using the Benjamini and Hochberg method (adjusted *P*-values ≤ 0.01). (**C**) Representative images and quantification of the percentage of cells immunofluorescent for C3 (red). Unpaired *t*-test. Three experimental repeats of two control and two *VCP* mutant lines as a minimum. (**D**) Immunofluorescence demonstrating GFAP-positive astrocytes with C3 immunolabelling in control and *VCP* mutant mouse spinal cords, *top* graph shows the C3-labelled proportion of GFAP-positive astrocytes. *Bottom* graph displays the percentage of GFAP-positive cells. Unpaired *t-*test. Three mice per group. Data-points for all of the immunolabelling quantification are the number of technical repeats (fields) used in the experiment. Data are presented as mean ± SEM. Scale bar = 20 μm. ns = not significant.

Having established that our hiPSC platform can recapitulate a reactive astrocyte state, we sought to determine whether familial ALS mutations were sufficient to induce cell-autonomous reactive transformation in the absence of extrinsic cues. We first excluded a mutation-dependent effect on astrocyte specification between control and *VCP* mutant cultures (*VCP*^R155C^ and *VCP*^R191Q^; Fig. 2A). Transcriptional profiling for genes associated with astrocytic reactivity^6^ confirmed differential expression in the *VCP* mutant compared to control astrocytes including increased expression of *HLA-E*, *CP*, *MX1* and a significant increase in expression of *HLA-A* (Fig. 2B). Although RNA-seq and quantitative PCR (Supplementary Fig. 2) did not show a significant increase of the *C3* transcript in *VCP* mutant astrocytes, a recognized marker of reactive transformation,^6^ using high throughput imaging and single cell analysis we observed a significant increase in basal C3 protein expression in *VCP* mutant astrocytes by qICC [*VCP* = 36.30 ± 2.714% (55 fields), control = 15.27 ± 1.431% (54 fields) *P* ≤ 0.0001; Fig. 2C]. We next sought to investigate whether C3 expression persisted at a later stage of the disease by examining *VCP*^A232E^ transgenic adult mouse tissue and indeed found GFAP-positive astrocytes in this context are C3-positive [*VCP*^A232E^ = 29.40 ± 2.418% (18 fields), *VCP*^WT^ = 13.56 ± 2.305% (18 fields), *P* ≤ 0.0001; Fig. 2D]. Together, these data demonstrate that *VCP* mutant hiPSC-derived astrocytes display hallmarks of reactive transformation.

### Isochronic *VCP* and *SOD1* mutant astrocytes exhibit divergent expression of reactivity markers

**Figure 3:**
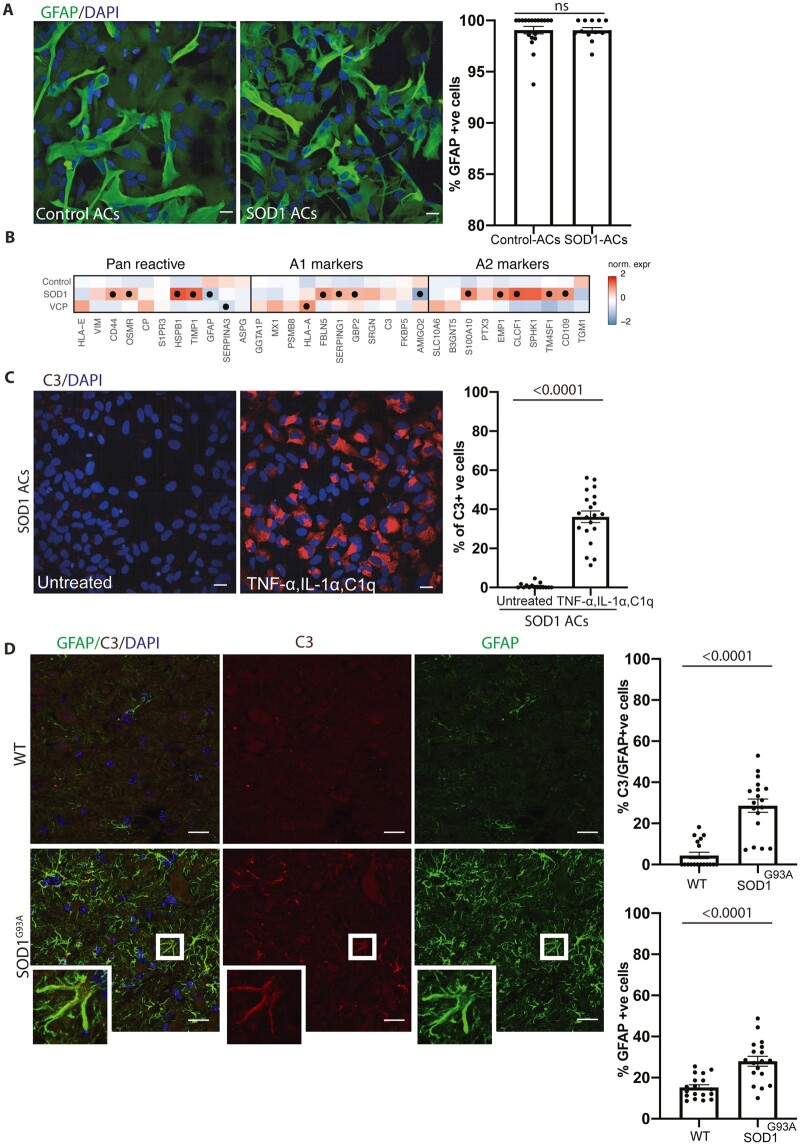
**Distinct reactive states in *VCP* and *SOD1* mutant astrocytes.** (**A**) Representative images and quantification of the percentage of cells immunofluorescent for GFAP (green). Mann-Whitney test. One experimental repeat of two lines per group. (**B**) Heat maps of pan reactive, A1 and A2 markers in control, *SOD1* and *VCP* astrocytes. Gene expression data represent SD from mean of the variance stabilized values across control, *SOD1* and *VCP* astrocytes. Points represent significantly increased transcripts as determined with the Wald test and following correction for multiple testing using the Benjamini and Hochberg method (adjusted *P*-values ≤ 0.01). (**C**) Representative images and quantification of the percentage of cells immunofluorescent for C3 (red). Mann-Whitney test. One experimental repeat of two lines per group. (**D**) Immunofluorescence demonstrating GFAP-positive astrocytes with C3 immunolabelling in wild-type (WT) and *SOD1* mutant mice, *top* graph shows the C3-immunolabelled proportion of GFAP-positive astrocytes. *Bottom* graphs display the percentage of GFAP-positive cells. Mann-Whitney test. Three mice per group. Data-points for all the immunolabelling quantification are the number of technical repeats (fields) used in the experiment. Data are presented as mean ± SEM. Scale bar = 20 μm (in **A** and **C**).

We next similarly interrogated isochronic cultures of hiPSC-derived *SOD1*^D90A^ (hereafter referred to as *SOD1*) mutant astrocytes. We first showed that the *SOD1* mutation again had no impact on astrocyte specification (Fig. 3A). However, RNA-seq revealed that *SOD1* mutant astrocytes also undergo reactive transformation but have distinct expression profiles of an array of reactivity-related markers compared to the *VCP* mutant astrocytes that included: pan reactive markers: *HSPB1*, *TIMP1*, *CD44* and *OSMR*; A1 markers: *SERPING1*, *FBLN5* and *GBP2*; and A2 markers: *S100A10*, *EMP1*, *TM4SF1* and *CD109* (Fig. 3B). Furthermore, analysis of hiPSC-derived *FUS* mutant astrocytes and publicly available *C9orf72* mutant astrocytes^15^ supported our findings of (i) cell autonomous reactive transformation in familial ALS astrocytes; and (ii) distinct profiles of reactivity markers (Supplementary Figs 3 and 4). Direct comparison between *VCP* and *SOD1* mutant hiPSC-derived astrocytes further demonstrated increased C3 expression in *VCP* compared to *SOD1* mutant astrocytes (*VCP*: 35.45 ± 3.043, *SOD1*: 21.67 ± 1.29, *P* = 0.0026; Supplementary Fig. 5). However, treating SOD1 mutant astrocytes with the aforementioned pro-inflammatory cues significantly increased C3 expression [untreated *SOD1* = 0.7 ± 0.343% (15 fields), treated *SOD1* = 36.14% ± 2.948 (20 fields), *P* < 0.0001; Fig. 3C]. We therefore reasoned that the difference in C3 expression under basal conditions might be explained by either a mutation-related temporal disparity in reactive transformation and/or the impact of neighbouring cells. To address this further, we examined *SOD1*^G93A^ transgenic adult mouse tissue and indeed found GFAP-positive astrocytes in this context are C3-positive [wild-type = 4.414 ± 1.566% (18 fields), *SOD1*^G93A^ = 28.59 ± 3.249% (18 fields), *P* ≤ 0.0001; Fig. 3D], consistent with recent studies.^16^ These findings demonstrate that although C3 expression is different in *SOD1* mutant hiPSC-derived astrocytes compared to those carrying *VCP* mutations, both sets of mutant astrocytes do ultimately undergo (C3-related) reactive transformation. Having demonstrated the presence of an innate reactive state in both *SOD1* and *VCP* mutant astrocytes, we next addressed whether mutant astrocytes could elicit C3 expression in control astrocytes, by treatment with their conditioned medium. We found no significant increase in C3 expression in control astrocytes on treatment with either *VCP* or *SOD1* mutant astrocyte conditioned media (Supplementary Fig. 6). These findings indicate that reactive *VCP* and *SOD1* mutant astrocytes do not induce C3 expression in healthy astrocytes.

### Molecular and functional evidence for distinct reactive states in *VCP* and *SOD1* mutant astrocytes

**Figure 4:**
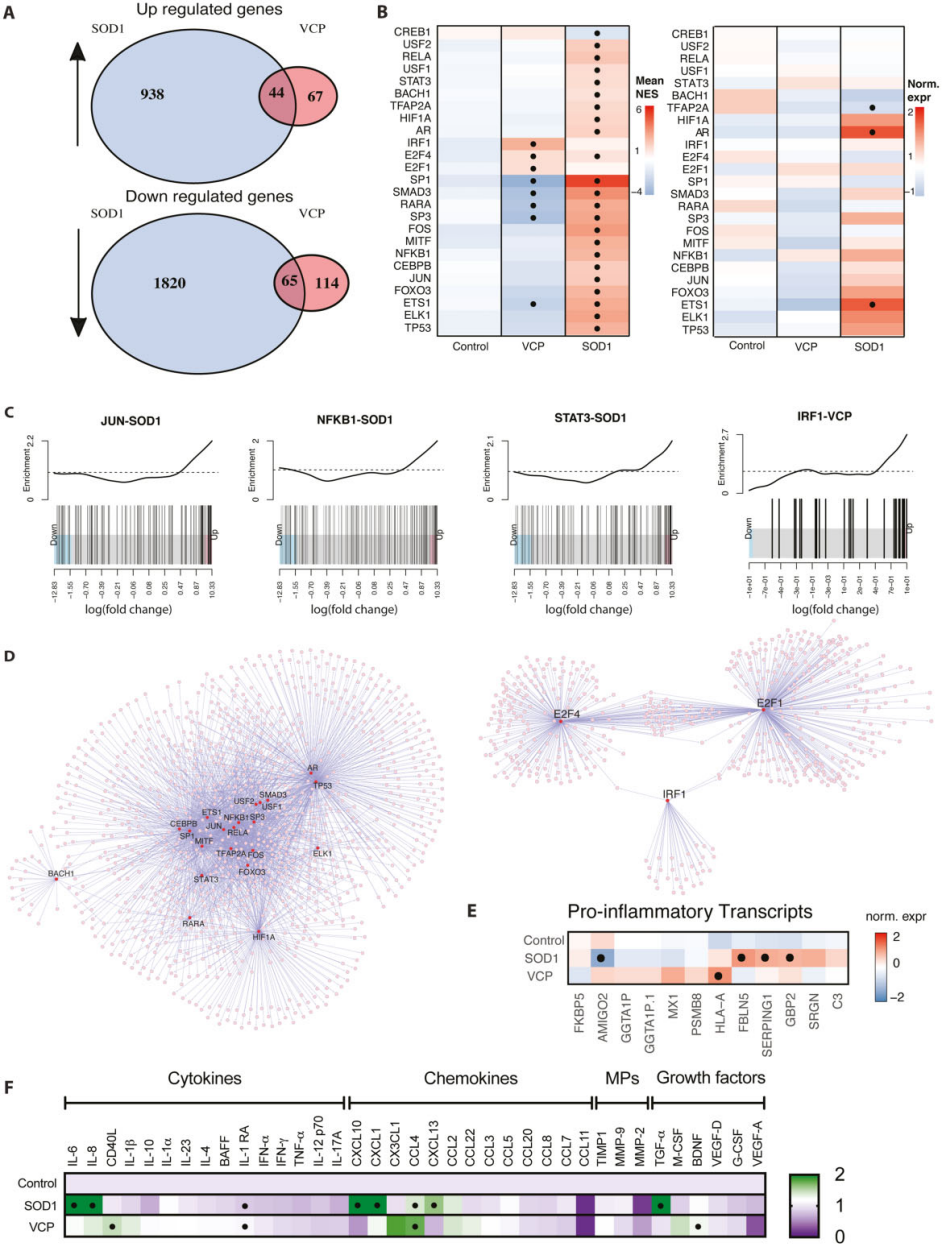
**RNA-seq and cytokine assay showing diversity of reactive changes in *SOD1* and *VCP* mutant astrocytes.** (**A**) Venn diagrams summarizing the up- and downregulated genes from the RNA-seq in *SOD1* and *VCP* mutant astrocytes. (**B**) Heat maps showing the mean normalized enrichment scores (NES) of indicated transcription factor regulons in *SOD1* and *VCP* mutant astrocytes (*left*), and inferred changes in gene expression of the same targets (*right*). In the *left* heat map the points represent significantly changed enrichments as assessed with a Student’s *t*-test (*P*-values ≤ 0.01). In the *right* heat map, gene expression data represent SD from mean of the variance stabilized values across control, *SOD1* and *VCP* samples, while points represent significantly increased transcripts as determined with the Wald test and following correction for multiple testing using the Benjamini and Hochberg method (adjusted *P*-values ≤ 0.01). (**C**) Barcode plots showing ranking and enrichments of the targets of the indicated transcription factors within the ordered lists of the differentially expressed genes for the indicated genetic background of astrocyte. (**D**) Transcription factor network plots based on the over-represented regulon enrichments in *SOD1* (*left*) and *VCP* (*right*) samples. Purple lines indicate edges and circles the nodes. Larger node size and red colour indicate candidate transcription regulators. (**E**) Heat map of pro-inflammatory transcripts in *SOD1* and *VCP* mutant astrocytes. Gene expression data represent SD from the mean of the variance stabilized values across control, *SOD1* and *VCP* samples. Points represent significantly increased transcripts as determined with the Wald test and following correction for multiple testing using the Benjamini and Hochberg method (adjusted *P*-values ≤ 0.01). (**F**) Luminex multiplex immunoassay (Thermo Fisher) showing the differentially released factors between control, *VCP* and *SOD1* mutant astrocytes. Data-points for the Luminex multiplex assay are the technical repeats (two wells/line) of one to two lines in each group, one-way ANOVA with *post hoc* Bonferroni-adjusted estimated marginal means tests. MPs = matrix metalloproteinases.

Supporting observations of diverse reactive states, RNA-seq revealed divergent gene expression signatures of *VCP* and *SOD1* mutant astrocytes (Fig. 4A). Over-represented gene ontologies associated with the *VCP* mutation were linked to the major histocompatibility complex (MHC) class II response, while ontologies linked to cell rearrangements and response to stimulus were associated with the *SOD1* mutation (Supplementary Figs 7 and 8). Differentially expressed genes common to both types of mutant astrocyte showed depletion of ontologies associated with nervous system development and cell–cell adhesion (Supplementary Fig. 9).

Divergent gene expression signatures between hiPSC-derived *VCP* and *SOD1* mutant astrocytes suggest genotype-specific gene regulatory networks are at play. To examine this further we performed analytic rank-based enrichment analysis^17^ using curated and signed regulons^18^ to identify differential activity of transcription factors in the separate mutant astrocyte populations. This revealed 25 transcription factors that were differentially active in either one or both ALS backgrounds (Fig. 4B, left). Changed activity was paralleled by significant changes in gene expression for certain candidates in the *SOD1* mutant astrocytes (e.g. *AR* and *ETS1*), although most were devoid of gene expression changes to imply post-transcriptional regulation (Fig. 4B, right). Independent rotation gene set tests^19^ using curated regulons from the TRRUST database^20^ orthogonally supported the differential activity of 16/25 candidates (Supplementary Table 2), and detailed the over-representation of associated regulons in the most altered genes of the mutant astrocyte gene expression signatures. Notably, the MHC class I associated transcription factor, IRF-1, was specifically activated in *VCP* mutant astrocytes to offer a ready explanation for differential expression of multiple related genes (Fig. 4C), while NFKB1, STAT3 and JUN were among the multiple overactive transcription factors in *SOD1* astrocytes with roles in the response to cytokines (Fig. 4C). Indeed, the pseudo-regulon of all transcription factors identified in the *SOD1* mutant astrocytes is enriched in gene ontology terms associated with inflammatory responses and cell-surface signalling pathways (Supplementary Fig. 10). Since highly significant (*P* = 0.000996) overactivation of NFKB1 was observed in *SOD1* astrocytes, we further investigated NFKB isoforms by qICC. In line with these findings, a significant increase of NFKB1 nuclear intensity, was observed by qICC in *SOD1* mutant astrocytes (*SOD1* = 827.848 ± 70.28, control = 601.52 ± 38.19 *P* = 0.0058), but not *VCP* mutant astrocytes (Supplementary Fig. 11). Meanwhile, nuclear translocation of the RELA isoform of NFKB was not increased in *SOD1* or *VCP* mutant astrocytes but was significantly increased in response to TNF-α, IL-1α and C1q in control astrocytes (treated control = 1.31326 ± 0.08, untreated control= 0.88838.32 ± 0.02, *P* = 0.0189) (Supplementary Fig. 12). Moreover, while *VCP*-associated transcription factors had limited regulon overlap, transcription factors overactive in *SOD1* mutant astrocytes form a tightly interconnected network with inflammatory response associated candidates at the core (Fig. 4D).

The aforementioned gene regulatory networks suggest differing inflammatory statuses between *VCP* and *SOD1* mutant astrocytes. Accordingly, we examined an array of pro-inflammatory transcripts in our RNA-seq data and found that these were both differentially expressed and variably associated with the two genotypes (Fig. 4E). This finding was supported by differential expression of pro-inflammatory transcripts in hiPSC-derived mutant *FUS* and publicly available mutant *C9orf72* astrocyte datasets^15^ (Supplementary Figs 3 and 4). To determine whether this differential expression of pro-inflammatory transcripts might subsequently lead to different functional attributes, we analysed the basal secretion of various cytokines, chemokines, metallopeptidases and growth factors from the mutant astrocytes. Although we observed some similar patterns of secreted factor release between *VCP* and *SOD1* mutant astrocytes including IL-1RA and CCL4, overall, there were striking differences in their basal release of several secreted factors including IL-6, CXCL10, BDNF and CD40L (Fig. 4F). Together this confirmed mutation-related functional heterogeneity of early astrocyte reactive states in familial ALS.

## Discussion

It is increasingly recognized that astrocytes undergo reactive transformation in the context of neurodegeneration.^6^ In disease states, astrocytes are likely to exhibit distinct gene expression signatures in a context-specific manner (e.g. determined by proximity to the focus of degeneration/injury and chronicity of disease).^21^ However, the cellular autonomy and molecular uniformity underpinning these astrocyte reactive transformation responses in the context of ALS have remained poorly understood. Although animal models have provided invaluable insight into issues of cellular autonomy in ALS, it is noteworthy that most of these studies have been performed in overexpression models, which do not convey the mutant protein at pathophysiological levels. There is additionally increasing recognition of interspecies differences between rodent and human astrocytes,^22^ arguing for greater use of human experimental systems as preclinical models. Beyond interspecific differences, hiPSC models bypass the need to overexpress the gene of interest and provide the opportunity to study molecular processes in enriched populations of target cells.^7^ This approach therefore provides an ideal platform to resolve issues of cellular autonomy, specifically whether astrocytes are capable of mounting deleterious reactive transformation in the absence of microglia, other immune cells or indeed neurons.

We have previously shown a decrease in the survival of *VCP* mutant astrocytes versus control.^12^ This is consistent with other studies using hiPSC-derived astrocytes to model familial ALS.^23^ Deleterious astrocyte reactive states are characterized by the loss of supportive function and/or gain of toxic function. A recognized marker for this deleterious astrocyte state is C3, which is initially expressed in the developing brain where it is required for synaptic pruning.^24^ C3 has been shown to be re-expressed in adult stages upon neuroinflammation, neurodegenerative disease and ageing.^6,25^ Recently, C3-positive astrocytes have been identified in post-mortem spinal cord and motor cortices of sporadic-, *C9orf72-* and *SOD1-*ALS cases, suggesting commonality in astrocyte activation between different causes of ALS.^16^ However, human post-mortem tissue represents the end point of disease and thus cannot address temporal heterogeneity in the establishment of astrocyte reactive states, for which hiPSC models have a distinct advantage.

Many astrocyte differentiation protocols from human pluripotent stem cells have used foetal bovine serum to generate astrocytes.^10^ However, exposure to serum has major effects on astrocytic reactive state.^26^ For this reason, we used a chemically defined method of astrocyte differentiation. After confirming that a reactive state could be induced in our hiPSC-derived astrocyte cultures by using established pro-inflammatory cues, we next sought to examine early molecular attributes of reactivity states in genetically divergent forms of familial ALS by analysing recognized markers.^6,8^ Crucially, these astrocytes were derived in an identical manner, thus ruling out potential differences in regional identity and ageing, which are both increasingly recognized to contribute to functional heterogeneity.^27^ These analyses first confirmed that both *SOD1* and *VCP* mutant astrocytes can undergo cell-autonomous reactive transformation. However, we also revealed that they do so in a strikingly different manner, suggesting that molecular heterogeneity in early astrocyte reactive states is mutation-related. We found more differentially expressed genes in mutant *SOD1* astrocytes compared with *VCP* astrocytes, which may or may not correlate with phenotypic severity. Functional studies of basal cytokine release further confirmed significant differences between *SOD1* and *VCP* mutant astrocytes and argue for more complex molecular and cellular processes governing at least the early phase of disease in familial ALS.

Astrocytes have been previously reported to act as antigen-presenting cells in Parkinson’s disease post-mortem brain tissue and cultured human astrocytes, triggered by α-synuclein accumulation leading to the activation of T cells.^28^*VCP* mutant hiPSC-derived astrocytes show a cell-autonomous expression of C3 in their basal state together with an increased expression of *HLA-A*, which belongs to MHC class I. One of the main regulators of MHC-1 expression is transcription factor IRF-1. MHC class I gene promoter activity in astrocytes was shown to be controlled entirely through a single enhancer, the MHC-IRF-E, to which IRF-1 binds in response to interferon-gamma stimulation.^29^ In our data, IRF-1 is activated in *VCP* mutant astrocytes, consistent with its recognized role in increasing *HLA-A* expression. This is further supported by our functional studies, which confirm the release of factors such as CCL4, BDNF and CD40L. These data cumulatively indicate the induction of a cell-autonomous immune response by *VCP* mutant astrocytes.

In *SOD1* mutant astrocytes, many cytokines released (including IL-6, IL-8 and CXCL1) are direct targets of pro-inflammatory transcription factor NFKB.^30^ This is consistent with both the increased activity of NFKB in our analysis (Fig. 4B), increased expression of the NFKB1 isoform (Supplementary Fig. 11) and cell-autonomous reactive transformation in *SOD1* mutant astrocytes. It is noteworthy that although we did not observe expression of C3 in *SOD1* mutant astrocytes to the same degree as *VCP* mutant or treated control astrocytes, its expression could be robustly induced by treatment with pro-inflammatory cytokines. This suggests that non-cell-autonomous mechanisms, such as microglia-released factors, may further contribute to C3 expression in this context.^16^ Furthermore, C3-positive astrocytes were found in the ventral spinal cords of disease manifesting mutant *SOD1* mice. These findings argue that C3 expression in *SOD1* mutant astrocytes is to some degree mediated through a non-cell-autonomous mechanism and/or is temporally regulated, and that the regulation could be distinct compared to *VCP* mutant astrocytes. Our findings of cell autonomous reactive transformation and striking early molecular and functional heterogeneity among patient-derived astrocytes from different forms of familial ALS may bear significance when considering astrocyte reactivity as a putative therapeutic target.

## Supplementary Material

awab328_Supplementary_Data

## Abbreviations

ALS: amyotrophic lateral sclerosis
hiPSC: human induced pluripotent stem cell
qICC: quantitative immunocytochemistry
