# Supplementary material for: Astrocytes display cell autonomous and diverse early reactive states in familial amyotrophic lateral sclerosis
Source: Brain. 2022 Jan 19;145(2):481–9. doi: 10.1093/brain/awab328 (PMC9014746; doi:10.1093/brain/awab328)
Supplement: awab328_Supplementary_Data [file awab328_supplementary_data.pdf]

Supplementary information

Astrocytes display cell autonomous and diverse early reactive states in familial amyotrophic lateral sclerosis

Taha[§], Clarke[§], Hall, Tyzack, Ziff, Greensmith, Kalmar, Ahmed, Alam, Thelin, Garcia, Helmy, Sibley*, Patani*

Supplementary methods

Derivation of Human Fibroblasts and hiPSCs

Dermal fibroblasts were cultured in OptiMEM +10% FCS medium. The following episomal plasmids were transfected for iPSC generation: pCXLE hOct4 shp53, pCXLE hSK, and pCXLE hUL (Addgene), as previously reported [1]. Details of the lines used in this study are provided in supplementary table 1. Three of the control lines used (control 2 and control 3, control 5 and control6) are commercially available and were purchased from Coriell (cat. number ND41866*C), ThermoFisher Scientific (cat. number A18945), Cedars Sinai (Cat.number CS02iCTR-NTn4), NIH (cat.number CRMi003-A) respectively.

Generation of hiPSC-derived astrocytes

All cell cultures were maintained at 37°C and 5% carbon dioxide. iPSCs were maintained on Geltrex (Life Technologies) with Essential 8 Medium media (Life Technologies), and passaged using EDTA. Generation of hiPSC-derived astrocytes was carried out based on a previously described protocol [2]. Briefly, after neural conversion (7 days in a chemically defined medium containing 1 μM Dorsomorphin (Millipore), 2 μM SB431542 (Tocris Bioscience) and 3.3 μM CHIR99021 (Miltenyi Biotec) neural precursors were patterned for 7 days with 0.5 μM retinoic acid and 1 μM purmorphamine, followed by a 4-day treatment with 0.1 μM purmorphamine. After a propagation phase (>60 days) with 10 ng/ml FGF-2 (Peprotech) and were terminally differentiated to astrocytes using BMP4 (10 ng/ml, R&D) and LIF (10 ng/ml, Sigma-Aldrich) Details of iPSC lines are provided in supplementary table 1.

Generation of hiPSC-derived motor neurons

Motor neurons were obtained as previously described [2]. Briefly, iPSCs were treated as above until after patterning neural precursors were treated with 0.1 μM Compound E (Enzo Life Sciences) to promote cell cycle exit.

Treatment of hiPSC-derived astrocyte cultures

Astrocytes were treated with i) human TNF-α (100 ng/ml; R&D 210-TA/CF), human IL-1α (100 ng/ml; Sigma-Aldrich), human C1q (1µg/ml; abcam), ii) astrocyte conditioned media added 50:50 with N2B27 base media for 6 days with media changes every 2 days or iii)motor neuron conditioned media added 50:50 with N2B27 and compound E for 3 days.

Animals, transgenic models and tissue processing

The following transgenic mouse lines were used, and were analyzed as different experimental groups: (i) Female *SOD1*^{G93A} mice [B6SJL-Tg(SOD1*G93A)1Gur/J, Jackson Laboratories], postnatal day 100-107 (n = 3 mice). (ii) wild-type C56BL/6-SJL mixed background (Jackson Laboratories) were used as control (n = 3 mice). (iii) Female 9 month old *VCP*^{A323E} (n=3 mice) and (iv) *VCP*^{WT} (n=3 mice) C57BL/6 (St. Jude Children's Research Hospital, Memphis, TN, USA) [3]. Mice were bred and maintained at the UCL Institute of Neurology in standard individually ventilated cages with up to three mice per cage, in a temperature and humidity controlled environment with a 12-h light/dark cycle and had access to drinking water and food ad libitum. Cages were checked daily to ensure animal welfare. Body weight was assessed regularly to ensure no weight loss. For tissue collection, animals were injected with terminal anaesthesia (pentobarbital sodium, Euthatal) and were transcardially perfused with 4% paraformaldehyde. The spinal cord was removed and post-fixed with 4% paraformaldehyde for one hour and then cryoprotected overnight with 30% sucrose; 10 or 20 µm serial transverse cryosections were cut for immunofluorescence staining.

Immunolabeling, imaging and image analysis

Samples were fixed in 4% paraformaldehyde and then blocked in 5% bovine serum albumin (BSA) in PBS, 0.3% Triton X-100. Primary antibodies were diluted as follows: rabbit anti C3 (Dako, A0063) 1:1000, chicken anti GFAP (Abcam, ab4674) 1:500, mouse anti Nestin (Millipore, MAB5326) 1:200, rabbit anti NFκB p65 (Cell Signaling, D14E12) 1:400, rabbit anti NFKB p105 (Thermo Fisher, PAS-85292) 1:400 and rabbit anti Vimentin (Cell Signaling, 5741) 1:200. Primary antibody incubation was performed overnight at 4°C in 5% BSA and 0.3% Triton X-100 in PBS, followed by incubation with secondary alexa fluor fluorescent antibodies (Thermo Fisher) for one hour at room temperature. 4′,6-diamidino-2-phenylindole (DAPI) was used as a nuclear counterstain (100 ng/ml). For iPSC cultures, images were acquired using the Opera Phenix High-Content Screening System (Perkin Elmer) with a 40x water objective as confocal z-stacks with z-step of 1µm and were processed as maximum projection. Cells were then analysed on a cell-by-cell basis using an automated pipeline on Columbus TM Image Data Storage and Analysis System (Perkin Elmer) software version 2.8.0. To control for cell density in per field analysis, fields with a total number of cells less than 30 or more than 100 cells were removed from the analysis. For mice spinal cord sections, slides were mounted with mounting media (Dako) and were imaged using a 710 Laser Scanning Confocal Microscope (Zeiss). At least 5 z-stack images were taken

per mouse (N=3 mice per genotype) from at least two spinal cord sections using the 40x objective and data was analyzed using Fiji.

RNA extraction and qPCR

The Promega Maxwell RSC simplyRNA cells kit including DNase treatment, alongside the Maxwell RSC instrument, was used for RNA extractions. The nanodrop was used to assess RNA concentration and the 260/280 ratio. Reverse transcription was performed using the Revert Aid First Strand cDNA Synthesis Kit (ThermoFisher Scientific) using random hexamers. qPCR was performed using the PowerUP SYBR Green Master Mix (ThermoFisher Scientific) and QuantStudio 6 Flex Real-Time PCR System (Applied Biosystems). Primers for C3 (Forward: GGGCTCGCTGGTGGTAAAAA; Reverse: AGACACCGGCGTAATCCTTC) and GAPDH (Forward: ATGACATCAAGAAGGTGGTG; Reverse: CATACCAGGAAATGAGCTTG) were used. Specific amplification was determined by melt curve analysis and agarose gel electrophoresis of the PCR products. Primer pairs with 90-110% efficiency were used and RT-minus samples were used as negative controls. Data was analysed using the *ddCt* method and is expressed as fold change over control group.

RNA-seq

Library preparation followed the Illumina TruSeq RNA Access library preparation kit as per the manufacturer's instructions; 100ng total RNA was first fragmented, cDNA generated using random priming during first and second strand synthesis; sequencing adaptors are ligated to the resulting double-stranded cDNA fragments. The coding regions of the transcriptome were then captured from this library using sequence-specific probes before a final round of PCR amplification and second strand digestion occurs to create the final library. This strand-specific protocol was used for library preparation and samples were barcoded and multiplexed prior to sequencing on a HiSeq 2500 platform. Control, *VCP, SOD1* and *FUS* RNA-seq libraries were generated according to this protocol. *C9ORF72* mutant astrocyte datasets were accessed from [4] using accession code GSE142730.

Mapping and Quality Control

Raw fastq files had Illumina sequencing adapters removed with Cutadapt (v.1.10) before being mapped to the genome (GRCh38) using HiSat2 (v.2.0.4 [5]) with options -p 8 and --dta. Assembled reads were sorted with samtools (v.1.3.1[6]) and subsequently mapped to the transcriptome (ensembl v91) using stringtie (v.1.3.3[7]) with options -p 8, -e and --rf. Gene-level reads were determined with Stringtie, and sample quality control performed with NGSCheckMate (v.1.0.0 [7,8]), fastqc (v.0.11.2), RSeQC's (v.2.6.4[9]) geneBody_coverage command, and Picard's (v.2.6.0) CollectRnaSeqMetrics command.

Differential Expression

Differential expression analysis was carried out using DESeq2 (v.1.22.1[9,10]) according to recommended package guidelines. In brief this involved gene-level Wald tests following shrunken estimation of sample dispersions and fold changes. All genes with at least one count in one sample were used in each analysis to satisfy DESeq2's statistical model. FDR threshold for significance was set at 0.01 and determined using the procedure of Benjamini and Hochberg. Summary plots were generated with the R packages ggplot2 (v.2.3.1), VennDiagram (v.1.6.20) and gplots (v.3.0.1). Gene ontology analysis was carried out using the GO enrichment Analysis command from the WGCNA (v.1.69) R package [4,11]. Included terms were filtered to Bonferroni P-values < 0.1, and overlapping terms collapsed using REVIGO [12].

Gene regulatory networks

High confidence (category A and B), signed human regulons from the DoRothEA gene set resource were used for calculating enriched transcription factor scores [13]. Regulons were curated based on various lines of evidence including literature resources, ChIP-seq peaks, binding site motifs and interactions inferred directly from gene expression. The regulon enrichment within each gene signature was determined using the analytic rank-based enrichment analysis algorithm from the virtual inference of protein-activity by enriched regulon (VIPER) package[14]. A null model based on sample permutations was incorporated to account for both the expected non-uniform distribution of the targets on the gene expression signature and the extensive co-regulation of gene expression. The minimum regulon size was set at 25 target genes, and the differential activity of each transcription factor was determined with a Student's t-test with threshold set at $p < 0.05$. Network plots were generated using the R package, RedeR (v.1.36). Additional rototation gene set tests to support transcription factor regulon enrichments were carried out using the roast function of edgeR (v.3.26.8) according to vignette guidelines, using 9999 rotations, and with false discovery rate set at 0.1[15,16]. The regulons used were collected from the TRRUST (v.2) database and limited to a minimal size of 25 target genes [17].

Griess assay

Release of nitric oxide from iPSC-derived astrocytes following exposure to inducers of inflammatory signalling was indirectly measured using a Griess assay. Media from cultures was collected and mixed in a 1:1 ratio with a modified Griess reagent (Sigma-Aldrich). Sodium nitrite standards (NaNO2) were prepared in media, and absorbances measured using a spectrophotometer at 540 nm.

Cytokine array

Supernatants of hiPSC-derived astrocyte cultures were analysed using the Procartaplex™ 39-PLEX, Human Cytokine/Chemokine assay (Thermofisher, Waltham, MA) using the manufacturer's instructions, as previously described [18]. Each experimental condition/technical repeat was analyzed in duplicate (i.e., two samples per experimental cell culture well). Plates were

analyzed on a Luminex 200 platform (Luminex Corporation, Austin, TX). Heat maps were produced using the fold increase of each cytokine from either control or untreated hiPSC-derived astrocytes. Statistical analysis was performed using R.

Statistical analysis

Data and graphs are presented as mean ± SEM. Graphpad prism 8/9 (Graph Pad Software) was used to generate graphs and to perform tests for distribution and statistical significance for immunostaining quantification and Greiss assay data. The type of statistical tests with p values are indicated in the figure legends. Any p value below 0.05 was considered to be statistically significant. Non-significant p-values were labelled as “ns” in the text or in figures where relevant.

Compliance with ethical standards

For hiPSC work, informed consent was obtained from all patients and healthy controls in this study. Experimental protocols were all carried out according to approved regulations and guidelines by UCLH’s National Hospital for Neurology and Neurosurgery and UCL Queen Square Institute of Neurology joint research ethics committee (09/0272). All animal experiments described in this study were carried out under licence from the UK Home Office, and were approved by the Ethical Review Panel of the Institute of Neurology. All experiments were carried out following the guidelines of the UCL Institute of Neurology Genetic Manipulation and Ethic Committees and in accordance with the European Community Council Directive of November 24, 1986 (86/609/EEC). Animal experiments were undertaken under licence from the UK Home Office in accordance with the Animals (Scientific Procedures) Act 1986 (Amended Regulations 2012) and were approved by the Ethical Review Panel of the Institute of Neurology.

Supplementary table 1. Details of iPSC lines used in this study

iPSC line	Mutation present	Age of Donor	Sex of Donor	Inheritance	Clinical presentation
CTRL 1	None	78	Male	N/A	N/A
CTRL 2	None	64	Male	N/A	N/A
CTRL 3	None	Fetal	Female	N/A	N/A
CTRL 4	None	51	Female	N/A	N/A
CTRL 5	None	51	Male	N/A	N/A
CTRL 6	None	Fetal	Male	N/A	N/A
MUT 1	VCP R155C	43	Female	Parent	lower limb onset, dementia
MUT 2	VCP R155C	43	Female	Parent	
MUT 3	VCP R191Q	42	Male	Parent	upper limb onset, dementia
MUT 4	VCP R191Q	42	Male	Parent	
MUT 5	SOD1 D90A	70	Female	Uncle	lower limb onset, no dementia symptoms
MUT 6	SOD1 D90A	50	Female	Parent	upper limb onset, no dementia symptoms
MUT 7	SOD1 D90A	50	Female	Parent	

Supplementary table 2: Differential activity of transcription factors in mutant astrocytes as assessed by two independent analyses.

Transcription Factor	p.value.viper.SOD1	p.value.viper.VCP	roast.analysis.support
SP1	6.49E-05	0.00179	Yes
FOS	0.00047	NA	Yes
MITF	0.000471	NA	
SMAD3	0.000493	0.0103	
ELK1	0.000945	NA	
NFKB1	0.000996	NA	Yes
USF2	0.00113	NA	
RARA	0.00114	0.00598	
TP53	0.00154	NA	Yes
RELA	0.00266	NA	Yes
FOXO3	0.00331	NA	
ETS1	0.00456	0.018	Yes
HIF1A	0.00614	NA	Yes
AR	0.00618	NA	Yes
CREB1	0.0106	NA	Yes
SP3	0.0164	0.0228	Yes
USF1	0.0177	NA	Yes
TFAP2A	0.0211	NA	Yes
E2F4	0.0214	0.0176	
STAT3	0.026	NA	Yes
CEBPB	0.0309	NA	

JUN	0.0346	NA	Yes
BACH1	0.0361	NA	
IRF1	NA	0.00158	Yes
E2F1	NA	0.0273	Yes

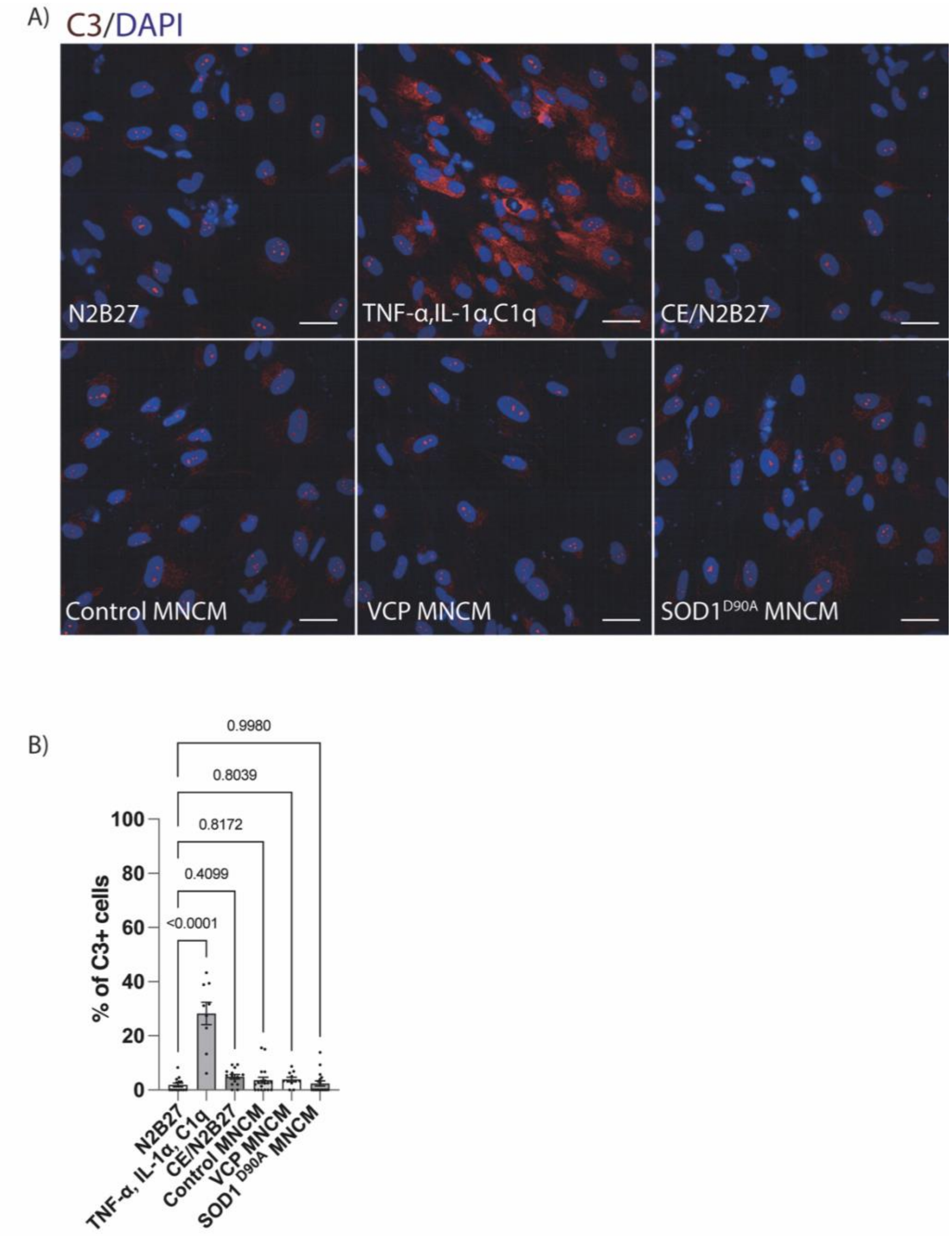

Figure S1: Motor neuron conditioned medium (MNCM) does not induce C3 expression in control astrocytes. (A) Representative images of C3 immunofluorescence (red) in astrocytes treated with basal media (N2B27), pro-inflammatory stimulation (with TNF-α, IL-1α and C1q), motor neuron basal media (Compound E and N2B27) and media taken from motor neuron cultures of *VCP* mutant and *SOD1*^{D90A}. (B) Quantification of the percentage of cells expressing C3 in response to different treatments of one control line, 2 technical repeats (wells) per condition. Data is shown as mean± SEM. Data points are technical repeats (fields) used in this experiment. *P*-values were obtained from One-way ANOVA followed by Dunnett's multiple comparison test. For all representative images, scale bar= 20 μm.

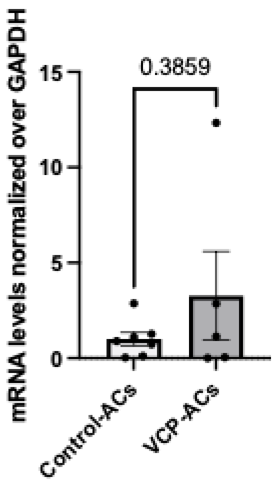

Figure S2: Measurement of C3 mRNA expression levels by qPCR. Bar graph showing C3 levels analyzed by qPCR in control and *VCP* mutant astrocytes and normalized over the housekeeping gene *GAPDH*. Data is expressed as fold change over control group mean. Data displayed as mean± SEM of 3 experimental repeats of 4 controls and 3 *VCP* mutant lines, with each datapoint representing the average across 2 technical replicates. *P*-value obtained from a Welch *t*-test.

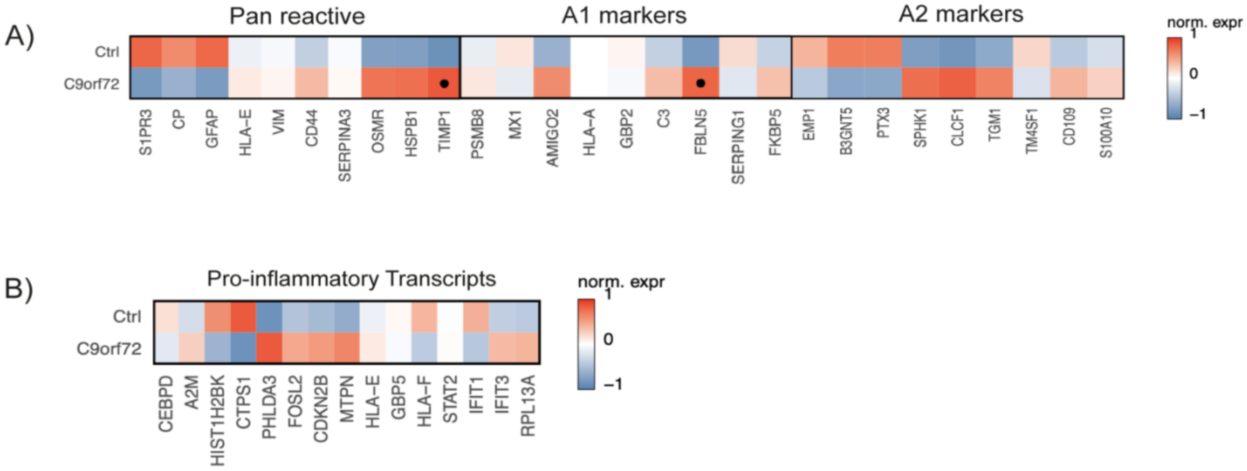

Figure S3: Transcriptional reactive profile of *C9ORF72* hiPSC-derived astrocytes (A) Heatmaps of Pan reactive, A1 and A2 markers in control, *C9ORF72* mutant astrocytes. Gene expression data represent SD from mean of the variance stabilised values across control and *C9ORF72* samples. Points represent significantly increased transcripts as determined with the Wald test and following correction for multiple testing using the Benjamini and Hochberg method (adjusted *p*-values ≤ 0.01. (B) Heat map of pro-inflammatory transcripts in control and *C9ORF72* mutant astrocytes. Gene expression data represent SD from mean of the variance stabilised values across control and *C9ORF72* samples. Points represent significantly increased transcripts as determined with the Wald test and following correction for multiple testing using the Benjamini and Hochberg method (adjusted *p*-values ≤ 0.01).

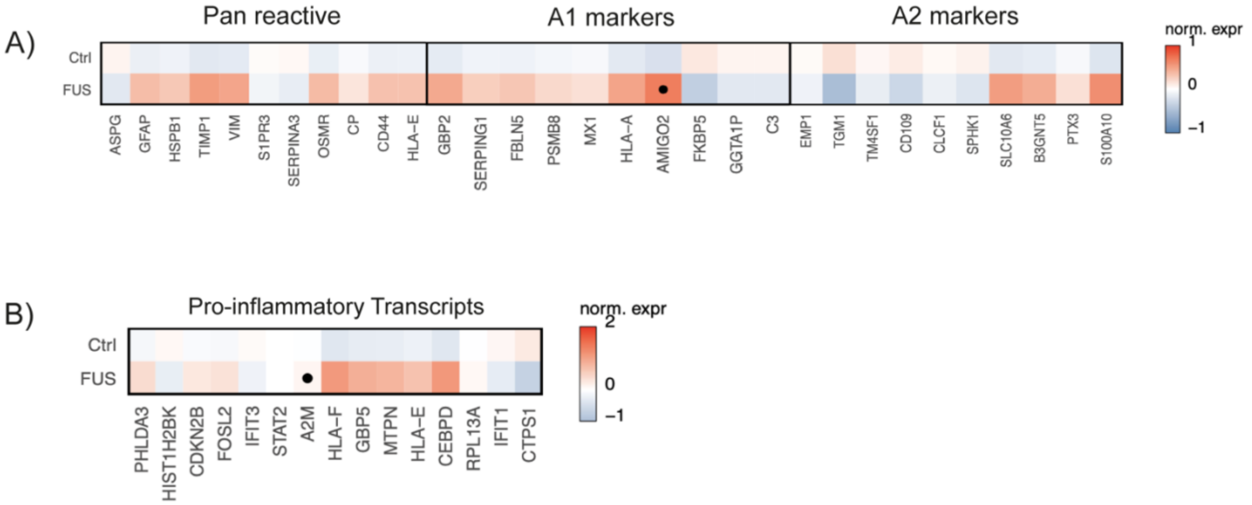

Figure S4: Transcriptional reactive profile of *FUS* hiPSC-derived astrocytes (A) Heatmaps of Pan reactive, A1 and A2 markers in control and *FUS* mutant astrocytes. Gene expression data represent SD from mean of the variance stabilised values across control and *FUS* samples. Points represent significantly increased transcripts as determined with the Wald test and following correction for multiple testing using the Benjamini and Hochberg method (adjusted p-values ≤ 0.01 (B) Heat map of pro-inflammatory transcripts in control and *FUS* mutant astrocytes. Gene expression data represent SD from mean of the variance stabilised values across control and *FUS* samples. Points represent significantly increased transcripts as determined with the Wald test and following correction for multiple testing using the Benjamini and Hochberg method (adjusted *p*-values ≤ 0.01).

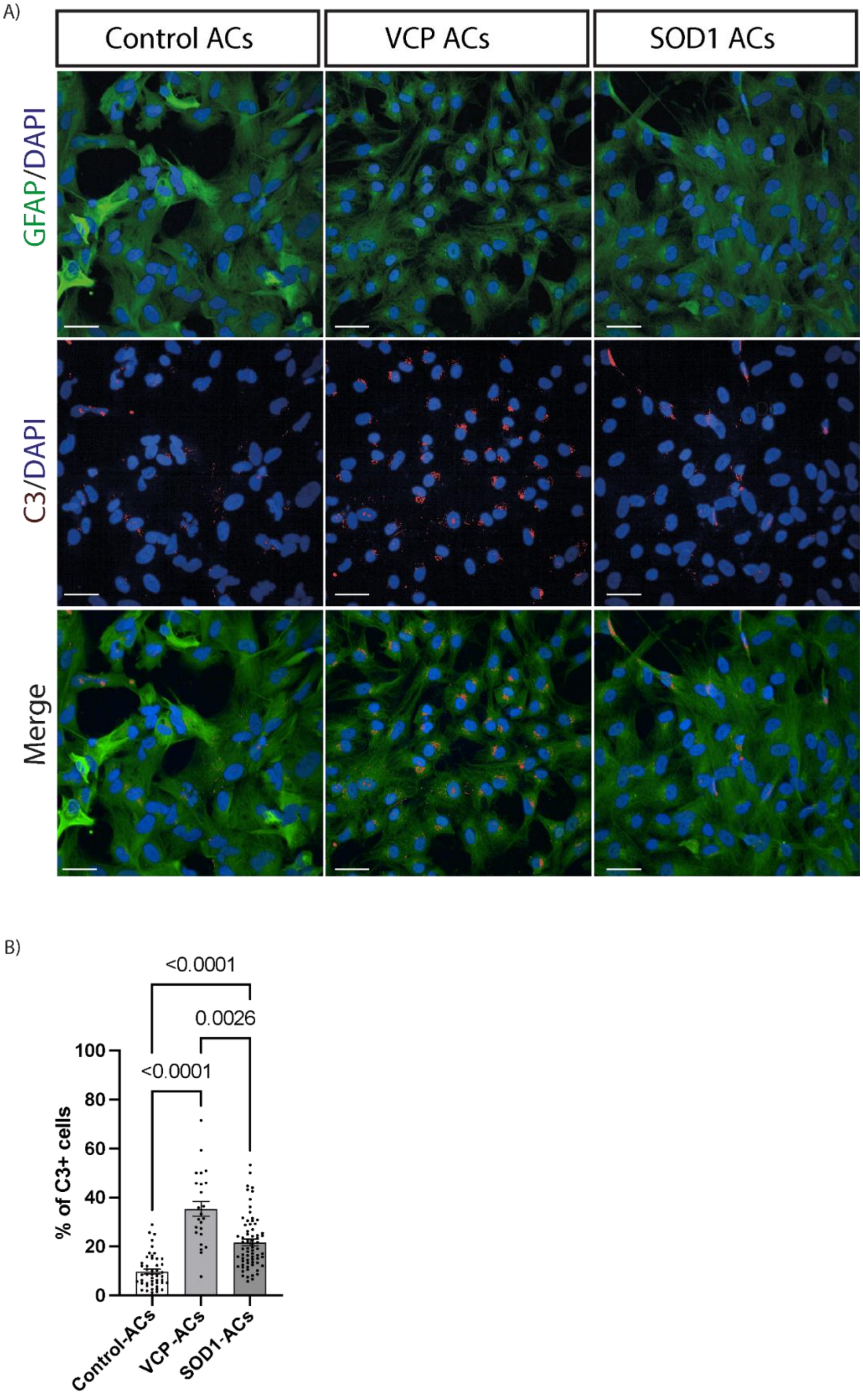
A)
Control ACs
VCP ACs
SOD1 ACs
GFAP/DAPI
C3/DAPI
Merge
B)
<0.0001
0.0026
<0.0001
% of C3+ cells
100
80
60
40
20
0
Control-ACs
VCP-ACs
SOD1-ACs

Figure S5: Differential expression of C3 positive astrocytes in mutant *SOD1* and *VCP* cultures. (A) Representative images of immunofluorescence staining for GFAP (green) and C3 (red) in control, mutant *VCP* and *SOD1* astrocyte cultures. (B) Quantification of the percentage of cells expressing C3 from 1-3 lines/group. Data is shown as mean ± SEM. Data points are technical repeats (fields) used in this experiment. *P*-values were obtained from One-way ANOVA followed by Dunnett's multiple comparison test. Scale bar: 20 μm.

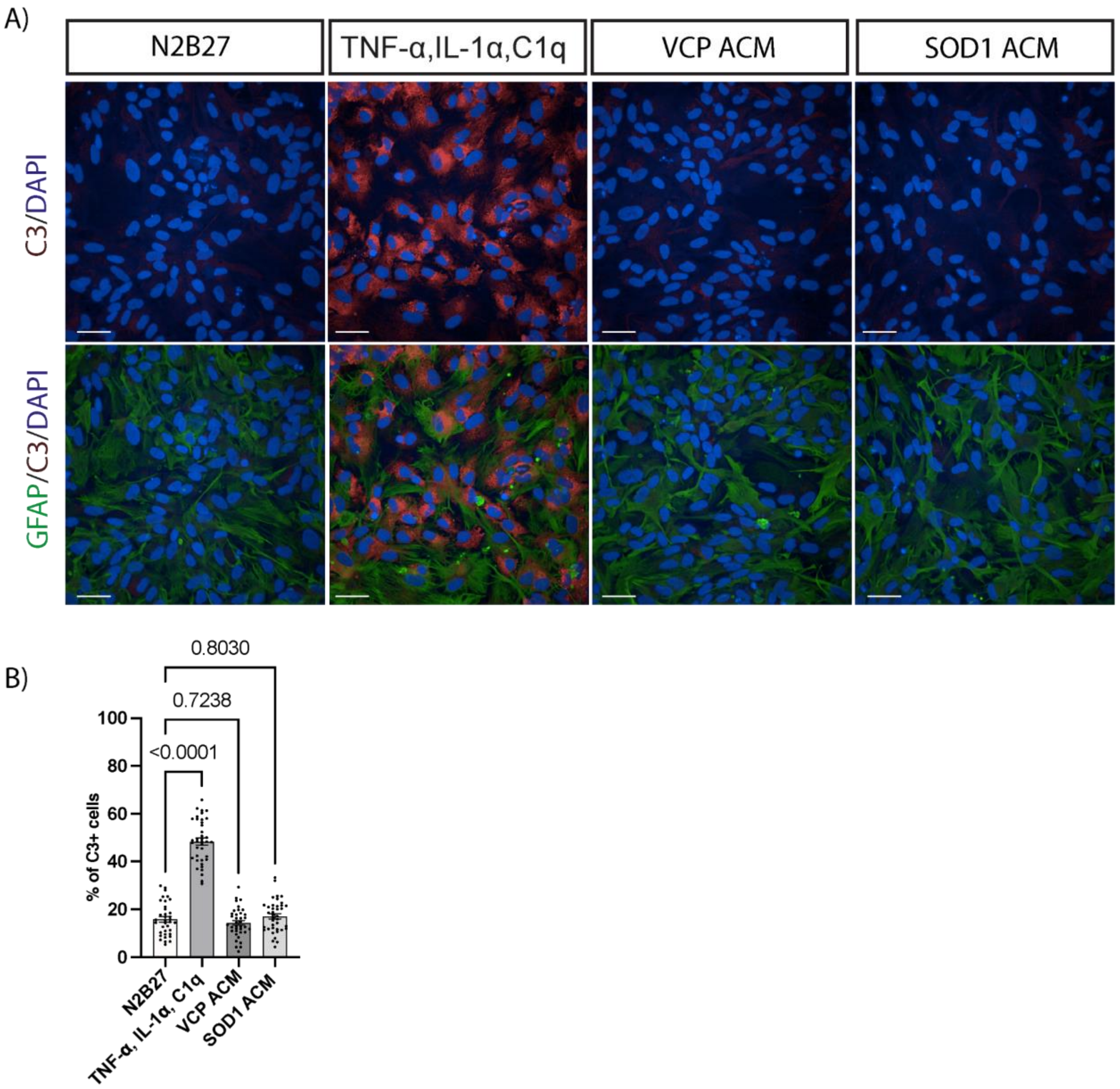

Figure S6: Effect of astrocyte conditioned medium (ACM) on control astrocytes reactivity. (A) Representative images of C3 immunofluorescence (red) in astrocytes treated with basal media (N2B27), proinflammatory stimulation (TNF-α, IL-1α and C1q), media taken from *VCP* and *SOD1* astrocyte cultures. (B) Quantification of the percentage of cells expressing C3 from 2 control lines. Data is shown as mean± SEM of 2 biological lines. Data points are technical repeats (fields) used in this experiment. *P*-values were obtained from One-way ANOVA followed by Dunnett's multiple comparison test. For all representative images, scale bar = 20 µm.

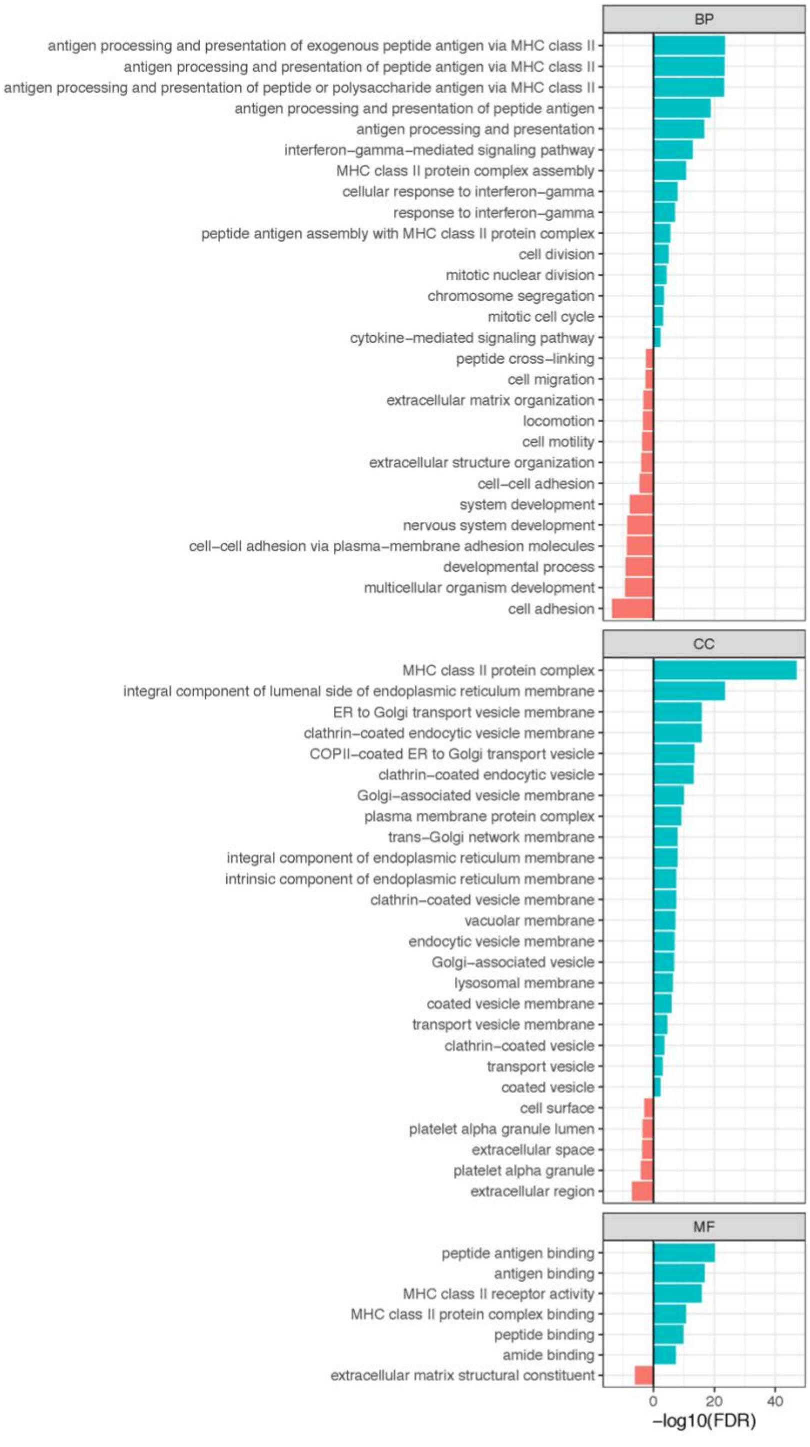

Figure S7: GO enrichment of *VCP* astrocytes. GO term analysis of differentially detected genes (adjusted p-values ≤ 0.01) in *VCP* mutant astrocytes. Shown are GO terms with Bonferoni corrected *p*-values <0.1.

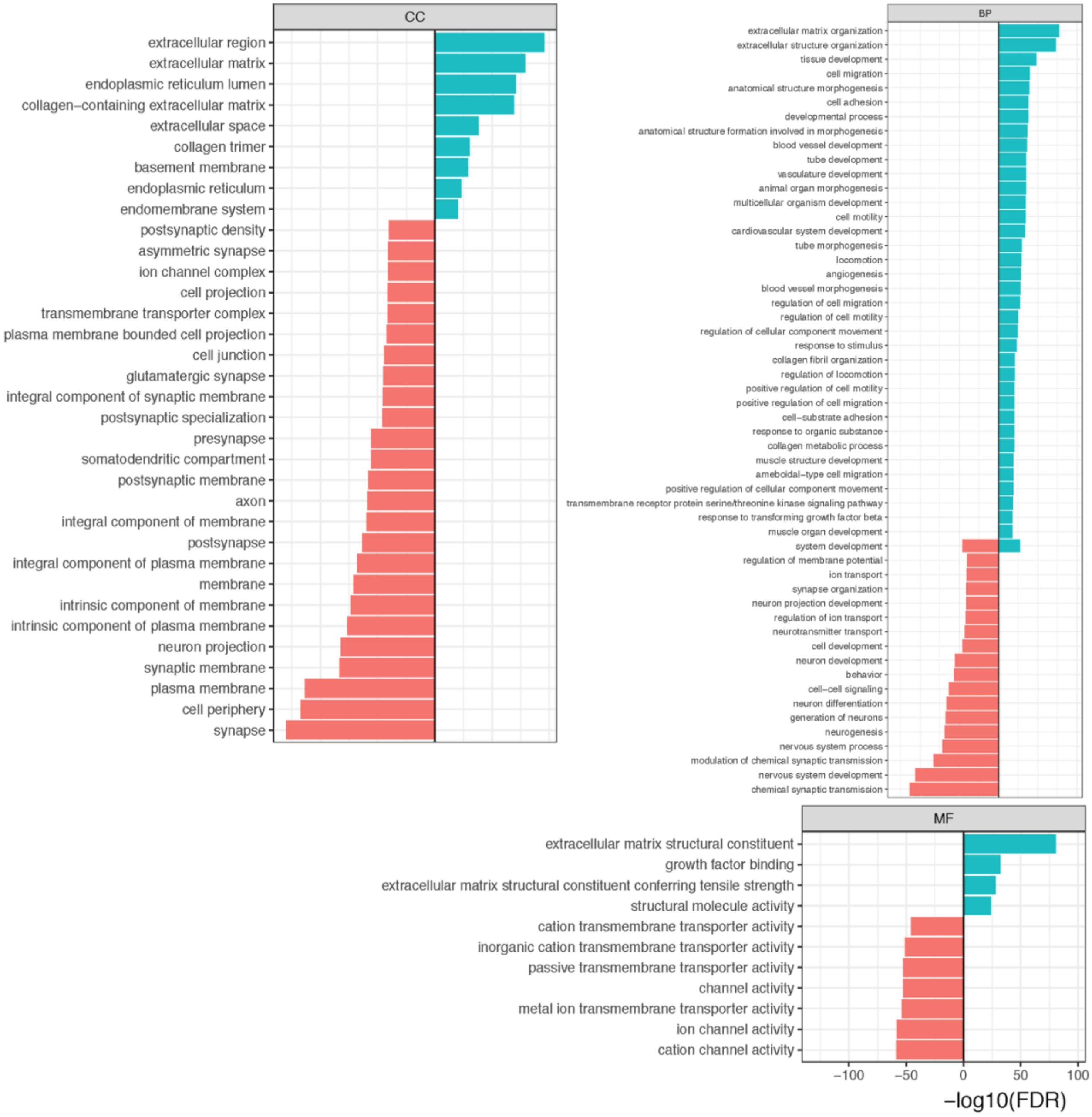

Figure S8: GO enrichment of *SOD1* astrocytes. GO term analysis of differentially detected genes (adjusted p-values ≤ 0.01) in *SOD1* mutant astrocytes. Shown are GO terms with Bonferroni corrected p-values <0.1.

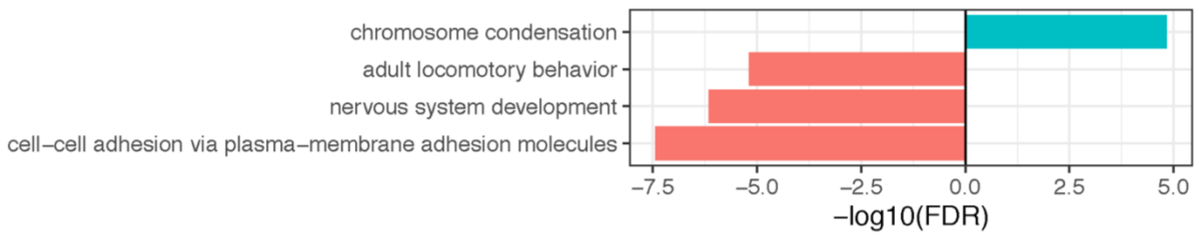

Figure S9: GO enrichment of common differentially expressed genes between *VCP* and *SOD1* astrocytes. GO term analysis of overlapping differentially expressed genes (adjusted *p*-values ≤ 0.01) in *SOD1* and *VCP* mutant astrocytes. Shown are GO terms with Bonferroni corrected *p*-values <0.1.

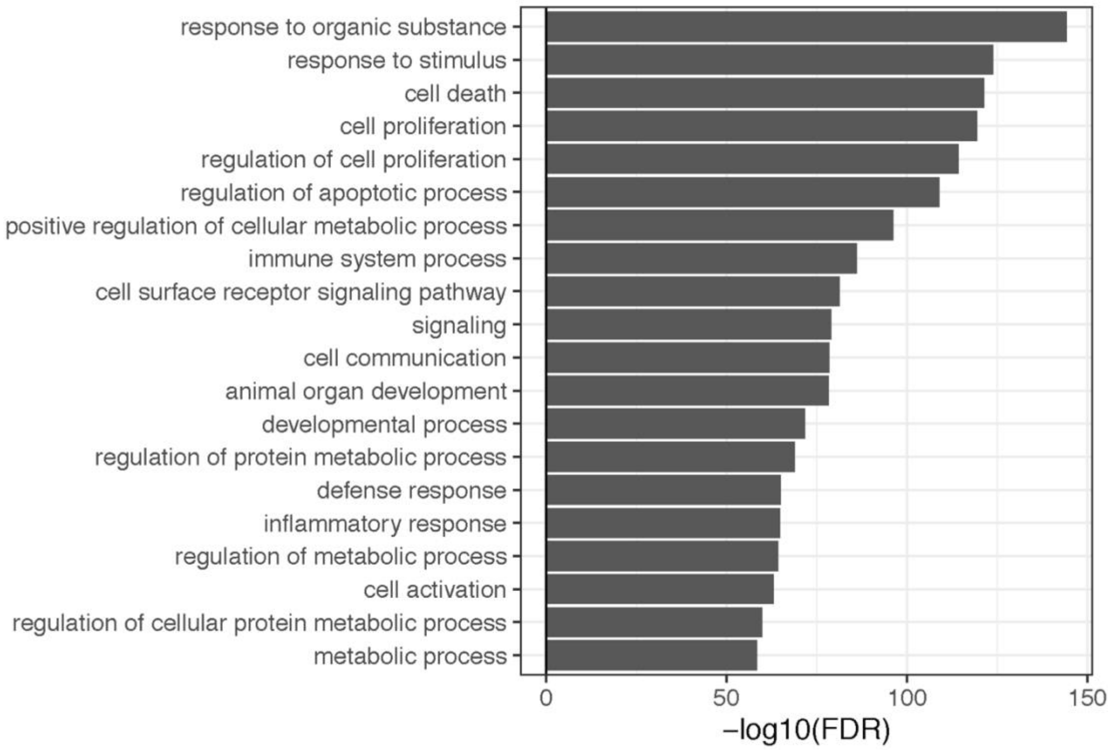

Figure S10: GO enrichment of transcription factors pseudo-regulon in *SOD1* astrocytes. GO term analysis showing the enriched genes of the transcription factor pseudo-regulon (adjusted *p*-values ≤ 0.01) in *SOD1* mutant astrocytes. Shown are GO terms with Bonferroni corrected *p*-values <0.1.

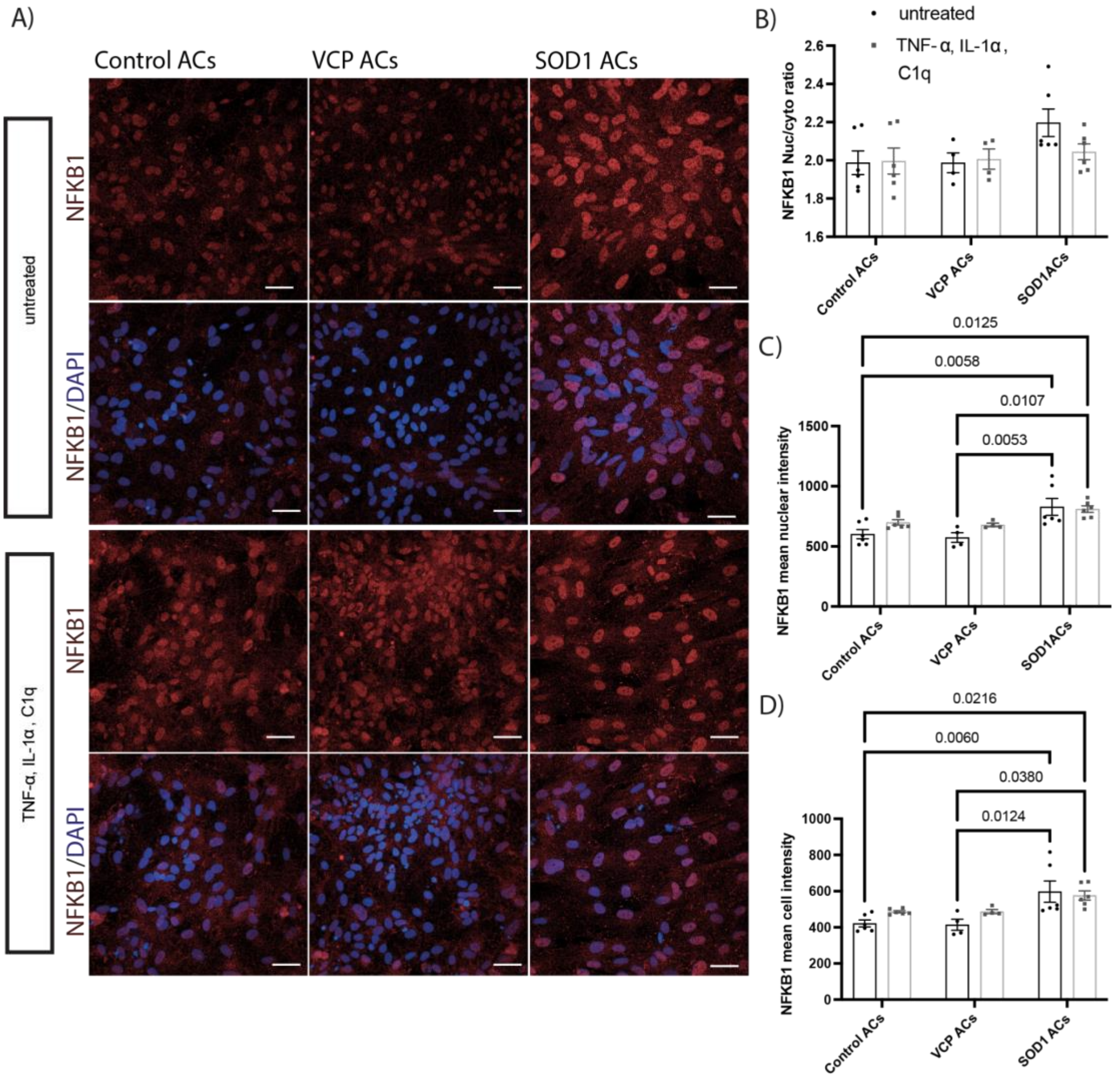

Figure S11: Increased nuclear NFKB1 expression in *SOD1* mutant astrocytes. (A) Representative images of NFKB1 immunofluorescence (red) in control, *VCP* and *SOD1* astrocytes treated with TNF-α, IL-1α and C1q. Quantification of (B) nuclear/cytoplasmic ratio, (C) mean nuclear intensity and (D) mean cell intensity for NFKB1 from 2-3 lines per group. Data is shown as mean ±SEM. Data points are technical repeats (wells) used in this experiment. *P*-values for (B and C) were obtained from 2-way ANOVA followed by Tukey's multiple comparison tests and for (D) was obtained from 2-way ANOVA followed by Sidak multiple comparison test. For all representative images, scale bar = 20 µm.

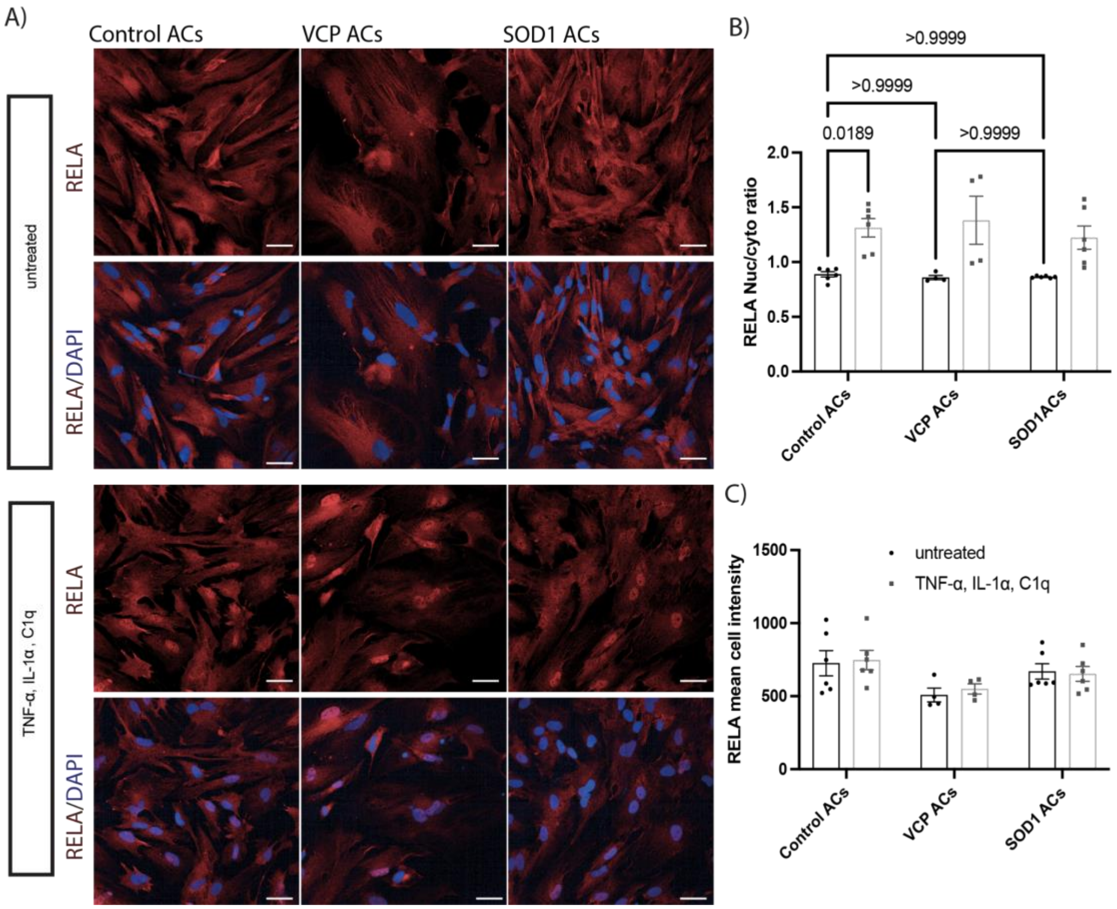

Figure S12: Increased nuclear RELA translocation in pro-inflammatory factor treated astrocytes. (A) Representative images of immunofluorescence staining for RELA (red) in control, mutant *VCP* and *SOD1* astrocytes treated with TNF-α, IL-1α and C1q. Quantification of (B) nuclear/cytoplasmic ratio and (C) mean cell intensity for RELA from 2-3 lines per group. Data is shown as mean ±SEM. Data points are technical repeats (wells) used in this experiment. *P*-values were obtained from 2-way ANOVA followed by Tukey's multiple comparison tests. For all representative images, scale bar= 20 µm.

Supplementary References:

1. Okita K, Matsumura Y, Sato Y, et al. A more efficient method to generate integration-free human iPS cells. *Nat Methods*. 2011;8(5):409-412.

2. Hall CE, Yao Z, Choi M, et al. Progressive Motor Neuron Pathology and the Role of Astrocytes in a Human Stem Cell Model of VCP-Related ALS. *Cell Rep*. 2017;19(9):1739-1749.

3. Custer SK, Neumann M, Lu H, Wright AC, Taylor JP. Transgenic mice expressing mutant forms VCP/p97 recapitulate the full spectrum of IBMPFD including degeneration in muscle, brain and bone. *Hum Mol Genet*. 2010;19(9):1741-1755.

4. Birger A, Ben-Dor I, Ottolenghi M, et al. Human iPSC-derived astrocytes from ALS patients with mutated C9ORF72 show increased oxidative stress and neurotoxicity. *EBioMedicine*. 2019;50:274-289.

5. Kim D, Langmead B, Salzberg SL. HISAT: a fast spliced aligner with low memory requirements. *Nat Methods*. 2015;12(4):357-360.

6. Li H, Handsaker B, Wysoker A, et al. The Sequence Alignment/Map format and SAMtools. *Bioinformatics*. 2009;25(16):2078-2079.

7. Pertea M, Kim D, Pertea GM, Leek JT, Salzberg SL. Transcript-level expression analysis of RNA-seq experiments with HISAT, StringTie and Ballgown. *Nat Protoc*. 2016;11(9):1650-1667.

8. Lee S, Lee S, Ouellette S, Park W-Y, Lee EA, Park PJ. NGSCheckMate: software for validating sample identity in next-generation sequencing studies within and across data types. *Nucleic Acids Res*. 2017;45(11):e103.

9. Wang L, Wang S, Li W. RSeQC: quality control of RNA-seq experiments. *Bioinformatics*. 2012;28(16):2184-2185.

10. Love MI, Huber W, Anders S. Moderated estimation of fold change and dispersion for RNA-seq data with DESeq2. *Genome Biol*. 2014;15(12):550.

11. Langfelder P, Horvath S. WGCNA: an R package for weighted correlation network analysis. *BMC Bioinformatics*. 2008;9:559.

12. Supek F, Bošnjak M, Škunca N, Šmuc T. REVIGO summarizes and visualizes long lists of gene ontology terms. *PLoS One*. 2011;6(7):e21800.

13. Garcia-Alonso L, Holland CH, Ibrahim MM, Turei D, Saez-Rodriguez J. Benchmark and integration of resources for the estimation of human transcription factor activities. *Genome Res*. 2019;29(8):1363-1375.

14. Alvarez MJ, Shen Y, Giorgi FM, et al. Functional characterization of somatic mutations in cancer using network-based inference of protein activity. *Nat Genet*. 2016;48(8):838-847.

15. Wu D, Lim E, Vaillant F, Asselin-Labat M-L, Visvader JE, Smyth GK. ROAST: rotation gene set tests for complex microarray experiments. *Bioinformatics*. 2010;26(17):2176-2182.

16. Robinson MD, McCarthy DJ, Smyth GK. edgeR: a Bioconductor package for differential expression

analysis of digital gene expression data. *Bioinformatics*. 2010;26(1):139-140.

17. Han H, Cho J-W, Lee S, et al. TRRUST v2: an expanded reference database of human and mouse transcriptional regulatory interactions. *Nucleic Acids Res*. 2018;46(D1):D380-D386.

18. Thelin EP, Tajsic T, Zeiler FA, et al. Monitoring the Neuroinflammatory Response Following Acute Brain Injury. *Front Neurol*. 2017;8:351.
